# A Novel Minimally-Invasive Method to Sample Human Endothelial Cells for Molecular Profiling

**DOI:** 10.1371/journal.pone.0118081

**Published:** 2015-02-13

**Authors:** Stephen W. Waldo, Daniel A. Brenner, James M. McCabe, Mark Dela Cruz, Brian Long, Venkata A. Narla, Joseph Park, Ameya Kulkarni, Elizabeth Sinclair, Stephen Y. Chan, Suzaynn F. Schick, Namita Malik, Peter Ganz, Priscilla Y. Hsue

**Affiliations:** 1 Division of Cardiology, San Francisco General Hospital, University of California San Francisco, San Francisco, CA, United States of America; 2 Division of Cardiology, San Francisco General Hospital, University of California San Francisco, San Francisco, CA, United States of America; 3 Division of Cardiology, San Francisco General Hospital, University of California San Francisco, San Francisco, CA, United States of America; 4 Division of Cardiology and the Center of Excellence in Vascular Research, San Francisco General Hospital, University of California San Francisco, San Francisco, CA, United States of America; 5 Division of Experimental Medicine, University of California San Francisco, San Francisco, CA, United States of America; 6 Division of Cardiology, San Francisco General Hospital, University of California San Francisco, San Francisco, CA, United States of America; 7 Division of Cardiology, San Francisco General Hospital, University of California San Francisco, San Francisco, CA, United States of America; 8 Division of Occupational and Environmental Medicine, University of California San Francisco, San Francisco, CA, United States of America; Centro Cardiologico Monzino, ITALY

## Abstract

**Objective:**

The endothelium is a key mediator of vascular homeostasis and cardiovascular health. Molecular research on the human endothelium may provide insight into the mechanisms underlying cardiovascular disease. Prior methodology used to isolate human endothelial cells has suffered from poor yields and contamination with other cell types. We thus sought to develop a minimally invasive technique to obtain endothelial cells derived from human subjects with higher yields and purity.

**Methods:**

Nine healthy volunteers underwent endothelial cell harvesting from antecubital veins using guidewires. Fluorescence-activated cell sorting (FACS) was subsequently used to purify endothelial cells from contaminating cells using endothelial surface markers (CD34 / CD105 / CD146) with the concomitant absence of leukocyte and platelet specific markers (CD11b / CD45). Endothelial lineage in the purified cell population was confirmed by expression of endothelial specific genes and microRNA using quantitative polymerase chain reaction (PCR).

**Results:**

A median of 4,212 (IQR: 2161 – 6583) endothelial cells were isolated from each subject. Quantitative PCR demonstrated higher expression of von Willebrand Factor (vWF, P<0.001), nitric oxide synthase 3 (NOS3, P<0.001) and vascular cell adhesion molecule 1 (VCAM-1, P<0.003) in the endothelial population compared to similarly isolated leukocytes. Similarly, the level of endothelial specific microRNA-126 was higher in the purified endothelial cells (P<0.001).

**Conclusion:**

This state-of-the-art technique isolates human endothelial cells for molecular analysis in higher purity and greater numbers than previously possible. This approach will expedite research on the molecular mechanisms of human cardiovascular disease, elucidating its pathophysiology and potential therapeutic targets.

## Introduction

The human endothelium is a key mediator of vascular homeostasis and overall cardiovascular health [1]. Endothelial dysfunction thus confers an adverse cardiovascular prognosis and plays a central role in the development of atherosclerosis and its clinical complications [2]. Sampling human endothelial cells from living subjects would potentially allow investigators to characterize the biology of the endothelium on a molecular level in both health and disease. Further, research focused on endothelial cells derived from human subjects could elucidate the molecular underpinnings of endothelial dysfunction while helping to identify novel biomarkers and potential therapeutic targets to mitigate the clinical manifestations of atherosclerosis [2].

Prior studies have described a method to isolate human endothelial cells from iliac arteries in patients undergoing angiography [3]. This methodology yielded an average of 262 endothelial cells and employed immunocytochemistry as well as single cell reverse transcription polymerase chain reaction (PCR) to analyze proteins and transcripts expressed at the site of vascular lesions. This minimally invasive procedure has yielded important insights into the role of the endothelium in various diseases, including heart failure and diabetes [4–6]. The technique has been limited, however, by low cellular yields and contamination of endothelial cells with other circulating cell types [4,7]. A standardized method to consistently produce a homogeneous population of human endothelial cells suitable for molecular studies would thus have significant utility. Accordingly, we report a novel technique to isolate human endothelial cells with a marked increase in yield and purity.

## Methods

### Population

The present study included healthy volunteers enrolled in a study involving smoke exposure at San Francisco General Hospital. Briefly, this cohort consisted of non-smoking healthy volunteers that were recruited to evaluate the cardiovascular effects of second-hand and third-hand cigarette smoke exposure. Each individual underwent endothelial cell sampling before and after exposure to smoke or clean air using a randomized, cross-over experimental design. All subjects provided written informed consent for this portion of the investigation and the study was approved by the Committee on Human Research at the University of California, San Francisco. Furthermore, this study was conducted under the guidelines specified in the Declaration of Helsinki.

### Endothelial Cell Sampling

Nine human volunteers underwent simultaneous peripheral blood and endothelial cell sampling from the antecubital vein. A 21-gauge peripheral intravenous catheter was placed in the antecubital vein under semi-sterile conditions using standard technique. Four 0.025” J-wires measuring 35 cm in length and constructed of stainless steel (Arrow International, Teleflex Incorporated, Research Triangle Park, NC) were sequentially inserted into the previously placed peripheral intravenous cannula. Each wire was advanced into the vessel and then withdrawn ten times while external compression was applied to the vein to increase contact between the wire and vessel wall. The wires were then inserted into a conical tube containing commercially available Cell Dissociation Buffer (Gibco, Life Technologies, Waltham, MA). The entire harvesting procedure was consistently completed within five minutes or less.

### Fluorescence-Activated Cell Sorting

**Fig 1:**
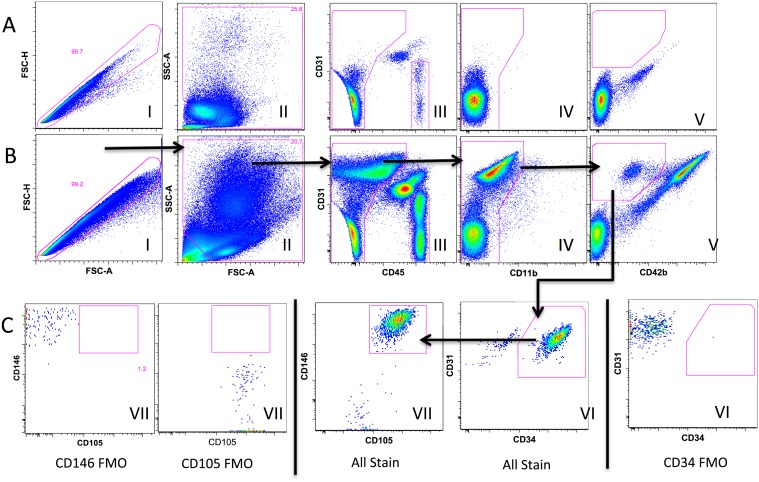
Fluorescence-activated cell sorting (FACS) Gating Strategy. The gating strategy to first eliminate leukocytes and platelets is shown on a representative endothelial biopsy sample (B) and a matched venous blood sample (A). Single cells are identified by plotting forward scatter-area against forward scatter-height (plot I); single cells are then separated from debris by a forward versus side scatter (plot II); leucocytes are eliminated by gating on CD45-negative (plot III) and CD11b-negative (plot IV) events; then platelets are eliminated by gating on CD42b-negative events (plot V). The brightly stained CD31+CD42b- events (plot V) shown in the endothelial biopsy sample (B) are absent from blood (A) indicating enrichment for CD31+ endothelial cells. Row C shows further enrichment of the CD31+ cells from an endothelial biopsy (All Stain) by positive selection with the endothelial markers, CD34 (plot VI), followed by CD146 and CD105 (plot VII). Endothelial biopsy samples were also stained with FMO controls for CD34 (plot VI), CD146 (plot VII) and CD105 (plot VII) to help set gates for sorting of the All Stain sample. Endothelial cells CD45-CD11b-CD42b-CD31+CD34+CD146+CD106+ (row C plot VII) and CD45+ lymphocytes (row B plot III) were sorted from endothelial biopsy samples.

Purification of the isolated cellular population was performed immediately after cell sampling. Cells adherent to the J-wires were removed via mechanical dissociation with a vortex at medium speed for 10 seconds. The resulting solution was subsequently centrifuged and the pellet was resuspended in phosphate buffered saline with 0.5% bovine serum albumin (BSA) and 2 mM ethylenediaminetetraacetic acid (EDTA) (Sigma-Aldrich, St. Louis, MO). The solution was then incubated with fluorescently-conjugated monoclonal antibodies to distinguish endothelial cells (CD45 - / CD11b- / CD42lo / CD31+ / CD34 + / CD105+ / CD146 +) from leukocytes (CD45+) and platelets (CD45- / CD42bhi) (Table 1) [8–13]. Following incubation, additional buffer was added and the sample was again centrifuged. The resulting cellular pellet was resuspended in 1 ml ammonium-chloride-potassium red cell lysis solution (Sigma-Aldrich) for five minutes at room temperature. After incubation, the cellular pellet was resuspended in 1 ml of buffer. Samples were subsequently sorted on a Becton-Dickinson FACS-Aria II according to the gating strategy illustrated in Fig. 1 to obtain endothelial cells and leukocytes. Controls included single stained beads (Life Technologies, Woburn, MA) for software-based compensation as well as whole venous blood processed and stained in parallel to assist with setting the anchor gates for each sort. The venous blood samples also served as a negative control, given the low number of isolated endothelial cells (Fig. 1, **Row A**). In addition, fluorescence-minus-one (FMO) controls were prepared on a subset of samples to demonstrate that staining with endothelial markers represented true antigen staining while also helping determine the proper location of the sorting gates to collect endothelial cells (Fig. 1, **Row C**). Cells were sorted into RNAse free tubes, pelleted immediately after sorting and then re-suspended in CellsDirect resuspension buffer with RNAse inhibitor (Life Technologies) before being store at -80°C until further analysis.

**Table 1:** Antigens and antibodies utilized for purification of human endothelial cells.

Antigen	Name	Clone	Fluorophore	Vendor
CD11b	Integrin Alpha M	ICRF44	BV-421	BioLegend
CD31	PECAM-1	WM59	Alexa-647	BD Biosciences
CD34	Glycoprotein for Cell Adhesion	581	PE-Cy7	BioLegend
CD42b	Glycoprotein 1b	HIP1	FITC	BD Biosciences
CD45	Protein Tyrosine Phosphatase	HI30	Alexa-700	Invitrogen
CD105	Endoglin, TGF Beta Receptor	266	PE-CF594	BD Biosciences
CD146	Melanoma Cell Adhesion Molecule	P1H12	PE	BD Biosciences

### RNA Extraction and Preparation

Gene expression was performed in the isolated endothelial cells and a leukocyte fraction (CD45+) isolated in parallel from the retrieved guidewires. As previously described, the number of freeze-thaw cycles was kept to a minimum in order to prevent degradation of mRNA and microRNA (miRNA) [14]. Total RNA extraction was performed using a MicroRNA Extraction Kit (Benevbio) according to the instructions supplied by the manufacturer. Reverse transcription was then performed to generate cDNA representing levels of either mRNA (High Fidelity cDNA Amplification Kit, Thermo Fisher Scientific/Life Technologies) or mature miRNA molecules (MicroRNA Assay Kit, Thermo Fisher Scientific/Life Technologies) [15]. Given the stem loop structures of the miRNA primers used for the initial reverse transcription, only mature miRNA molecules were amplified into cDNA. The reliability of the mRNA and miRNA extraction data was tested by quantifying the expression levels of the housekeeping gene β-actin [AB 4331182 (Hs99999903_m1)] and the small RNA, RNU48 [AB 4427975 (001006)] using specific RNA primers for amplification [16–18]. To allow detection of messenger transcript derived from a small number of cells, pre-amplification of cDNA representing levels of mRNA was performed (Taqman PreAmp Master Mix Kit, Thermo Fisher Scientific/Life Technologies) per the instructions supplied by the manufacturer.

### Quantification of mRNA and miRNA

After pre-amplification, Real Time Quantitative PCR (RT-QPCR) was utilized according to the two-step method for quantifying the levels of the candidate mRNA and miRNA in each sample. In the first step to generate cDNA constructs representing endogenous mRNA transcripts, random hexamers were used to conduct first-strand cDNA synthesis reactions for genes known to be differentially expressed in endothelial cells and leukocytes: von Willebrand Factor (vWF) [AB 4331182 (Hs01109446_m1)], nitric oxide synthase 3 (NOS3) [AB 4331182 (Hs01574659_m1)], vascular cell adhesion molecule 1 (VCAM-1) [AB 4331182 (Hs00365485_m1)] and CD45 [AB 4331182 (Hs04189704_m1)]. Then, an Applied Biosystems 7900HT Fast Real Time PCR device was used to amplify cDNA using a fluorescently labeled Taqman probe and primer set (Thermo Fisher Scientific/Life Technologies). Similarly, for quantifying the expression of an endothelial-enriched microRNA, microRNA-126 [19], we used a miR-126-specific stem loop-based RT primer for amplification as a first cDNA synthesis step. Subsequently, an Applied Biosystems 7900HT Fast Real Time PCR device was used to amplify such cDNA using a fluorescently labeled Taqman probe and primer set specific for miR-126 [AB 4427975 (002228)] (Thermo Fisher Scientific/Life Technologies). Raw Ct values were measured which represent the “real-time” cycle count at which the Taqman probe fluorescence increases exponentially. Relative expression (or fold-change) was calculated using the formula 2^(–ΔΔCt)^, as described previously [16]. The reliability of mRNA and miRNA extraction was supported by additional measurement of the housekeeping gene β-actin, which was used as the reference control for mRNA quantification, and the small RNA RNU48, which was used as the reference control for miRNA quantification. Amplicon sequences for each RT-QPCR reaction are listed in Table 2.

**Table 2:** Amplicon sequences for primers analyzing endothelial cell-specific gene expression and mature microRNA sequences for primers analyzing microRNA expression.

Gene	Amplicon/Mature Sequence
CD45 (PTPRC)	5’-GTGACAGGGCAAAGCCCAACACCTTCCCCCACTGGCCATCTGCAAGCTGAGGAGCAA-3’
Nitric Oxide Synthase 3 (NOS3)	5’- AGCAGGATGGGCCCTGCACCCCAAGACGCTGCCTGGGCTCCCTGGTATTTCCACGGAAACTACAGGGCCGGCCCTCCCCCGGCCCCCCGGCCCCTGAGCAGCTGCTG-3’
Vascular Cell Adhesion Molecule 1 (VCAM-1)	5’- CCAGAGATACAACCGTCTTGGTCAGCCCTTCCTCCATCCTGGAGGAAGGCAGTTCTGTGAAT-3’
von Willebrand Factor (vWF)	5’- ACCCTTTGTGCAGAAGGAACTCGCGGCAGGTCATCCACGGCCCGATGCAGCCTTTT-3’
miR-126	5’- UCGUACCGUGAGUAAUAAUGCG-3
β-Actin (ACTB)	5’-GGCGTGATGGTGGGCATGGGTCAGAAGGATTCCTATGTGGGCGACGAGGCCCAGAGCAAGAGA-3’
RNU48	5’- GATGACCCCAGGTAACTCTGAGTGTGTCGCTGATGCCATCACCGCAGCGCTCTGACC-3’

### Statistical Analysis

All data is presented as medians (intraquartile ranges, IQR) for non-normally distributed data and frequency (percentage) for categorical data. Gene expression data are presented as bar graphs representing the mean fold change compared to a reference with error bars representing the standard error of the mean (SEM). Comparison of mRNA and miRNA values were made between the endothelial sample and leukocyte sample fraction via a two-tailed, non-paired t-test. A p-value < 0.05 was considered statistically significant.

## Results

The median age of the nine healthy subjects studied was 46 years (IQR: 34–49) and 67% were male. Each of the subjects tolerated the endothelial cell sampling procedure well without any complications.

### Cell Sorting

The multi-parametric gating scheme effectively isolated endothelial cells from the other cell populations found in peripheral blood (Fig. 1). Compared to leukocytes and platelets, endothelial cells were larger and more granular based on forward and side-scatter profiles. Leukocytes and platelets were first excluded based on expression of CD45, CD11b and CD42b while endothelial cells were then identified based on expression of endothelial cell surfaces markers CD31, CD34, CD105 and CD146. The median cellular yield using this seven surface marker specific isolation method was 4,212 (IQR: 2161–6583) cells per subject.

### Gene Expression

**Fig 2:**
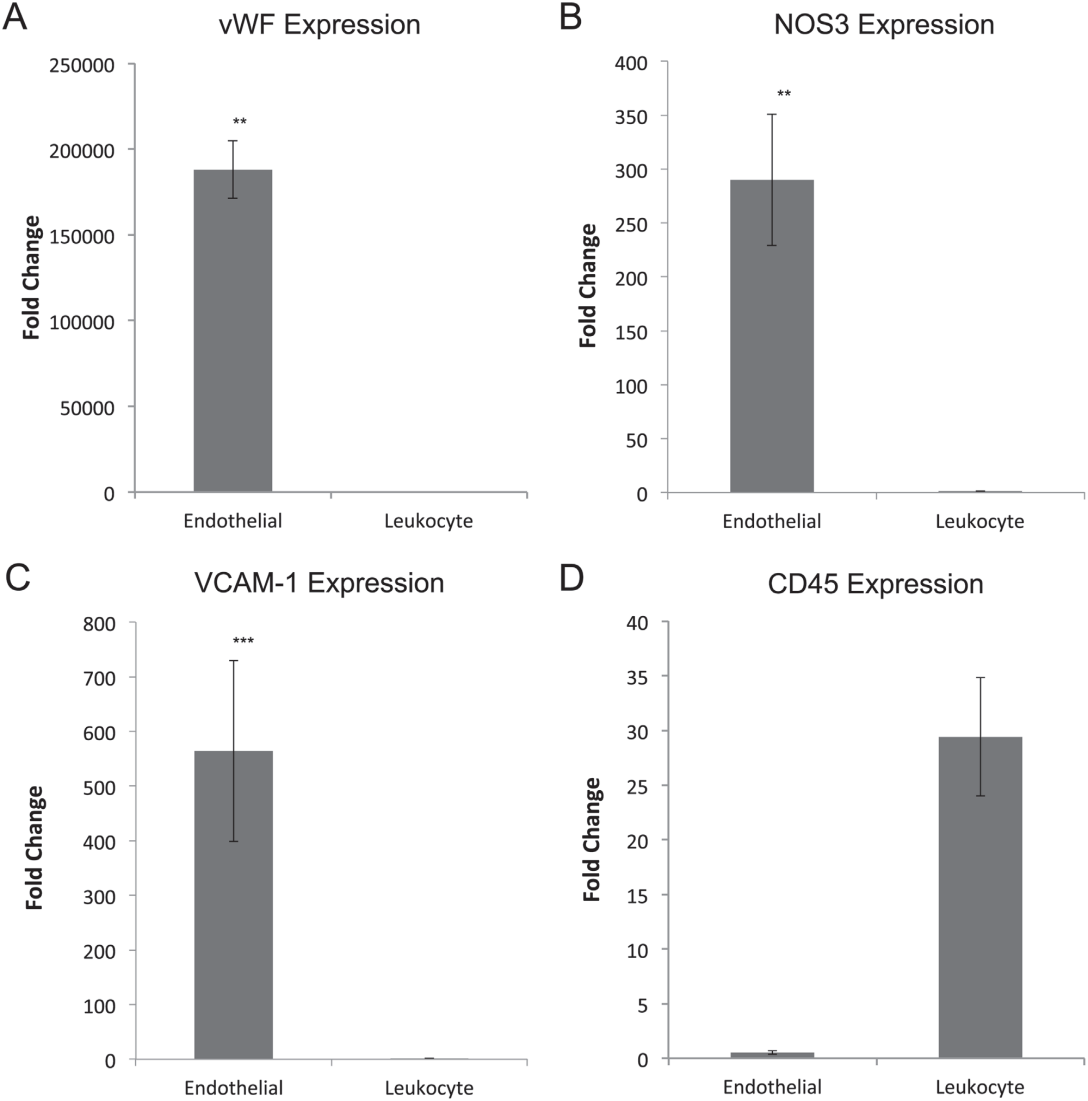
Endothelial cell molecular profiling (mRNA). Expression of endothelial specific genes such as (**A**) von Willebrand Factor (vWF), (**B**) nitric oxide synthase 3 (NOS3) and (**C**) vascular adhesion molecule 1 (VCAM-1) are significantly greater in the endothelial cell fraction compared to the leukocyte fraction. In contrast, expression of a leukocyte marker (CD45) (**D**) was numerically greater in the leukocyte cell fraction when compared to the endothelial faction (P = 0.101). Messenger RNA expression levels are referenced to β–actin. All values are mean fold change with the bars representing the standard error. ** Indicates P<0.001. *** Indicates P<0.003.

**Fig 3:**
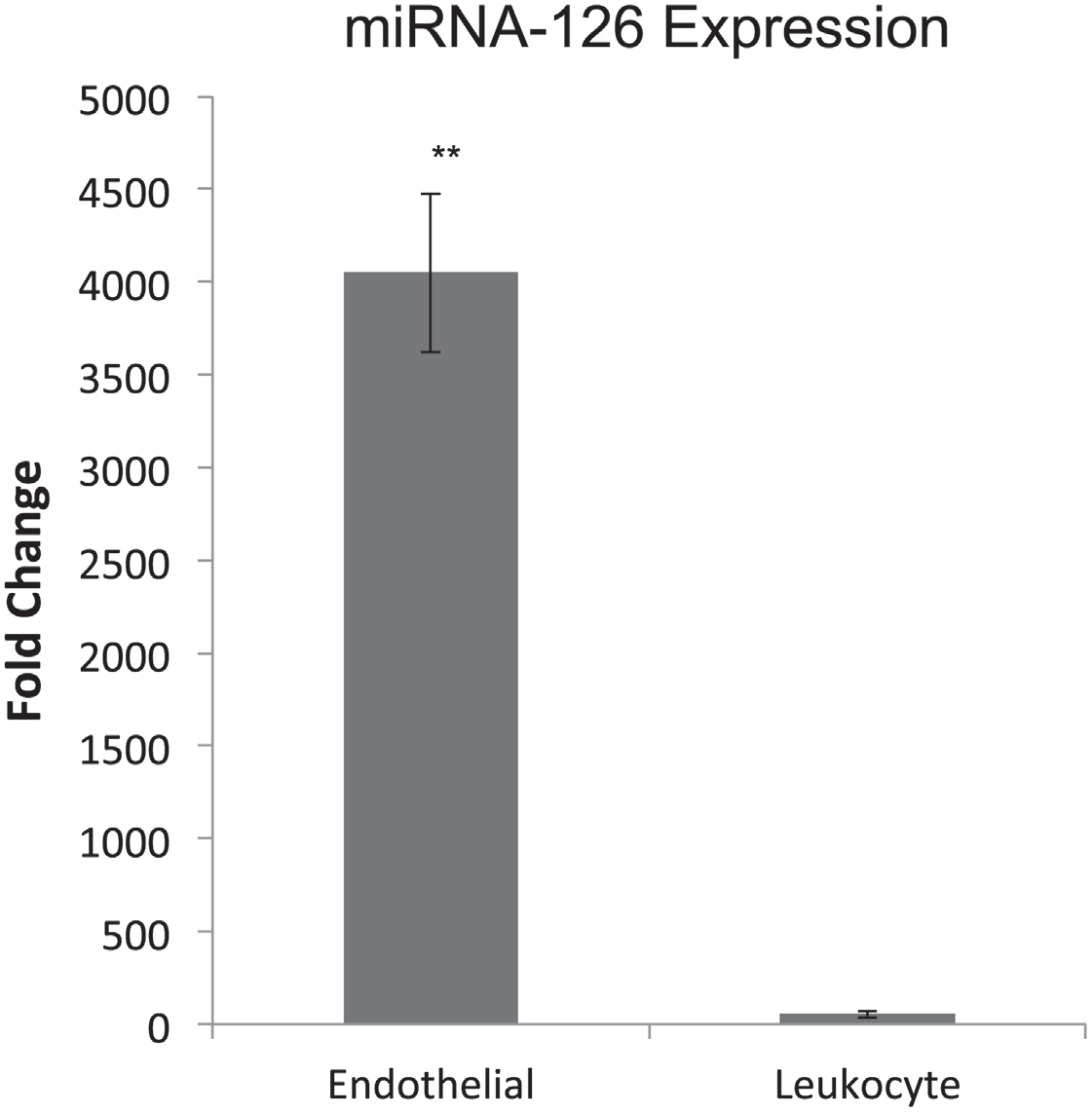
Endothelial cell molecular profiling (microRNA). Expression of endothelial specific microRNA-126 was significantly greater in the endothelial cell fraction when compared to the leukocyte faction. MicroRNA expression levels are referenced to RNU48. All values are mean fold change with the bars representing the standard error. ** Indicates P<0.001.

Gene expression profiling of the isolated cell fractions demonstrated an endothelial rich population, when compared to leukocytes isolated from guidewires from the same subjects in parallel (Fig. 2). Quantitative PCR demonstrated significantly increased expression of vWF (P<0.001), NOS3, (P<0.001) and VCAM-1 (P<0.003) in the purified endothelial population without expression of CD45 characteristic of leukocytes, though this did not reach statistical significance likely due to the small sample size (P = 0.101, **D**). In addition to these traditional mRNA endothelial markers, we also demonstrated enrichment of the endothelial cell-specific miRNA-126 in the isolated endothelial fraction as compared to the leukocyte fractions (P<0.001) (Fig. 3).

## Discussion

The present study describes a novel minimally-invasive technique to harvest and purify large quantities of homogeneous human endothelial cells suitable for molecular analysis. As the data demonstrate, this optimized approach results in endothelial cell yields that are twenty-fold greater than previously reported and with favorable purity, enabling analysis of gene expression and gene regulation. Future investigations employing this technique could elucidate the unique molecular mechanism underlying endothelial dysfunction in a variety of cardiovascular disease states thus leading to development of biomarkers and potentially the identification of novel therapeutic targets.

Fifteen years ago, Feng et al first described a technique for isolating human endothelial cells from iliac arteries [3]. Since this initial report, this methodology has been widely employed but often limited by low numbers of endothelial cells, contaminated with peripheral blood cells. In the present study, we report a revised technique to harvest human endothelial cells from an antecubital vein, a simple procedure that carries no additional risk above placing a peripheral intravenous catheter and can be easily performed in the outpatient setting or incorporated into any more invasive endovascular procedure. Subsequent isolation and purification with multi-parameter FACS using endothelial specific markers leads to a sufficient quantity of endothelial cells which allow for analysis of gene, protein and metabolite expression, studies which all require a high level of cellular homogeneity [20]. This technique also removes contaminating cells, such as leukocytes or platelets, that may interact with the endothelial cells of interest and alter their measured expression profiles *ex vivo* [21].

The wide application of this easily performed and highly adaptable technique could lead to a better understanding of endothelial cell physiology and pathophysiology. Previous research has suggested that gene expression in venous endothelial cells correlates well with expression in arterial endothelial cells, reinforcing the systemic nature of endothelial dysfunction [22]. Venous sampling as opposed to arterial sampling has thus become standard [4,23]. Endothelial cell sampling could now be performed when a peripheral intravenous line is placed in the outpatient or inpatient setting to garner a better understanding of systemic endothelial health. The technique is flexible enough, however, to also be performed in the arterial circulation when access is obtained for endovascular procedures, such as peripheral or coronary angiography. Simultaneous sampling of the arterial and venous endothelium in such a patient could advance our understanding of the similarities and differences in endothelial gene expression throughout the vasculature, furthering the investigation of human endothelial cell heterogeneity. The purification process is also easily adaptable as additional monoclonal antibodies could be included to identify surface antigens of interest. Cellular fixation and permeabilization steps could be added to the protocol to identify intracellular antigens, such as NF- κB, or post-translational protein modifications such as phosphorylation of eNOS. Alternatively, the cellular subpopulation could be subsequently used for quantitative analysis of any mRNA or miRNA of interest. Through this approach, one could gain a better appreciation of endothelial function and its contribution to human disease.
