# Correction: A novel minimally-invasive method to sample human endothelial cells for molecular profiling

**DOI:** 10.1371/journal.pone.0126062

**Published:** 2015-05-04

**Authors:** Stephen W. Waldo, Daniel A. Brenner, James M. McCabe, Mark Dela Cruz, Brian Long, Venkata A. Narla, Joseph Park, Ameya Kulkarni, Elizabeth Sinclair, Stephen Y. Chan, Suzaynn F. Schick, Namita Malik, Peter Ganz, Priscilla Y. Hsue

There are errors in the author affiliations. The affiliations should appear as shown here:

Stephen W. Waldo^1^, Daniel A. Brenner^2^, James M. McCabe^3^, Mark Dela Cruz^4^, Brian Long^5^, Venkata A. Narla^4^, Joseph Park^6^, Ameya Kulkarni^7^, Elizabeth Sinclair^5^, Stephen Y. Chan^6^, Suzaynn F. Schick^8^, Namita Malik^8^, Peter Ganz^4^, Priscilla Y. Hsue^4^

^1^ Currently, Division of Cardiology, Department of Medicine, Massachusetts General Hospital, Boston, MA, ^2^ Currently, Division of Cardiovascular Medicine, Stanford University Medical Center, Stanford, CA, ^3^ Currently, Division of Cardiology, Department of Medicine, University of Washington, Seattle, WA ^4^ Division of Cardiology and the Center of Excellence in Vascular Research, San Francisco General Hospital, University of California, San Francisco, CA, ^5^ Division of Experimental Medicine, University of California, San Francisco, CA, ^6^ Division of Cardiovascular Medicine, Brigham and Women’s Hospital, Boston, MA, ^7^ Currently, Division of Cardiology, Mid-Atlantic Permanente Medical Group, ^8^ Division of Occupational and Environmental Medicine, University of California, San Francisco, CA

Additionally, the following information is missing from the Funding section: Schick SF and Malik N were supported by the University of California Tobacco-Related Disease Research Program (20PT-0184).
